# Functional Characterization of a Spectrum of Novel Romano-Ward Syndrome *KCNQ1* Variants

**DOI:** 10.3390/ijms24021350

**Published:** 2023-01-10

**Authors:** Susanne Rinné, Annemarie Oertli, Claudia Nagel, Philipp Tomsits, Tina Jenewein, Stefan Kääb, Silke Kauferstein, Axel Loewe, Britt Maria Beckmann, Niels Decher

**Affiliations:** 1 Institute of Physiology and Pathophysiology, Vegetative Physiology, University of Marburg, 35037 Marburg, Germany; 2 Institute of Biomedical Engineering, Karlsruhe Institute of Technology (KIT), 76131 Karlsruhe, Germany,; 3 Department of Medicine I, University Hospital, LMU Munich, 80802 Munich, Germany; 4 Deutsches Zentrum für Herz-Kreislauferkrankungen (DZHK), Partner Site Munich, 80636 Munich, Germany; 5 Member of the European Reference Network for Rare, Low Prevalance and Complex Diseases of the Heart (ERN GUARD-Heart), 81377 Munich, Germany; 6 Institute of Surgical Research at the Walter-Brendel-Centre of Experimental Medicine, University Hospital, LMU Munich, Marchioninistrasse 27, 81377 Munich, Germany; 7 Institute of Legal Medicine, Goethe University, University Hospital Frankfurt, 60590 Frankfurt, Germany; 8 Institute for Transfusion Medicine and Immunohematology, German Red Cross Blood Service Baden-Württemberg-Hessen, Goethe University Frankfurt, 60528 Frankfurt, Germany; 9 Deutsches Zentrum für Herz-Kreislauferkrankungen (DZHK), Partner Site Frankfurt, 60596 Frankfurt, Germany

**Keywords:** potassium channel, KCNQ1, KvLQT1, LQTS, Romano-Ward syndrome

## Abstract

The *KCNQ1* gene encodes the α-subunit of the cardiac voltage-gated potassium (Kv) channel KCNQ1, also denoted as Kv7.1 or KvLQT1. The channel assembles with the ß-subunit KCNE1, also known as minK, to generate the slowly activating cardiac delayed rectifier current *I*_Ks_, a key regulator of the heart rate dependent adaptation of the cardiac action potential duration (APD). Loss-of-function variants in *KCNQ1* cause the congenital Long QT1 (LQT1) syndrome, characterized by delayed cardiac repolarization and a QT interval prolongation in the surface electrocardiogram (ECG). Autosomal dominant loss-of-function variants in *KCNQ1* result in the LQT syndrome called Romano-Ward syndrome (RWS), while autosomal recessive variants affecting function, lead to Jervell and Lange-Nielsen syndrome (JLNS), associated with deafness. The aim of this study was the characterization of novel *KCNQ1* variants identified in patients with RWS to widen the spectrum of known LQT1 variants, and improve the interpretation of the clinical relevance of variants in the *KCNQ1* gene. We functionally characterized nine human *KCNQ1* variants using the voltage-clamp technique in *Xenopus laevis* oocytes, from which we report seven novel variants. The functional data was taken as input to model surface ECGs, to subsequently compare the functional changes with the clinically observed QTc times, allowing a further interpretation of the severity of the different LQTS variants. We found that the electrophysiological properties of the variants correlate with the severity of the clinically diagnosed phenotype in most cases, however, not in all. Electrophysiological studies combined with *in silico* modelling approaches are valuable components for the interpretation of the pathogenicity of *KCNQ1* variants, but assessing the clinical severity demands the consideration of other factors that are included, for example in the Schwartz score.

## 1. Introduction

The slowly activating delayed rectifier potassium current (*I*_Ks_) is, together with the rapidly activating delayed rectifier potassium current (*I*_Kr_), responsible for the late phase of repolarization of the cardiac action potential (AP). The stimulation of cardiac beta-adrenergic receptors increases heart rate and contractility, and shortens the AP duration (APD), by activating *I*_Ks_. Voltage-gated channels that conduct *I*_Ks_ are formed by KCNQ1 (Kv7.1, KvLQT1) subunits, which assemble to form a tetrameric channel, together with single transmembrane domain containing KCNE1 ß-subunits, also denoted as minK [1,2]. All members of the KCNE family (KCNE1-5) assemble with KCNQ1 and diversely modify channel characteristics, with KCNE1 as the major cardiac accessory subunit of KCNQ1 [3,4].

The congenital Long-QT-syndrome (LQTS) is an inherited channelopathy, manifested in a prolonged QT-interval and arrhythmias caused by delayed repolarization [5], with a prevalence of about 1:2000 [6]. The electric abnormalities can lead to runs of *torsades de pointes* ventricular tachycardia, eventually resulting in syncope or even in sudden cardiac death. *KCNQ1* is the most commonly affected gene in LQTS patients [7,8,9]. Jervell and Lange-Nielsen syndrome (JLNS) is a rare autosomal recessive LQTS form, caused by *KCNQ1* variants [10,11,12,13]. Homozygous or biallelic compound variants affecting function can cause JLNS, characterized by congenital deafness combined with syncopal attacks and sudden death, due to prolonged QTc interval [10]. However, there are also patients with autosomal recessive LQT1 syndrome, genetically showing homozygous or biallelic missense compound variants, who do not suffer from hearing loss [11,14]. Additionally, there is the autosomal dominant form of LQTS1, also denoted as Romano-Ward Syndrome (RWS), usually a clinically milder form with genetically heterozygous missense, nonsense, exon skipping, and frameshift variants affecting KCNQ1 channel function [13,15].

The present study focuses on the electrophysiological characterization of nine *KCNQ1* variants associated with LQTS. Seven of them (G119R, K241E, L273V, R539L, delF166, delG186_L187, and G430fs*28) are described for the first time, while two (V254L and R591C) were previously reported [7,16,17,18]. Using voltage-clamp experiments in *Xenopus laevis* oocytes, we functionally characterized each variant in the homozygous and heterozygous state, in the presence and absence of KCNE1, and found that the severity of the loss-of-function found by electrophysiological experiments mostly correlates with the clinically diagnosed phenotype. Although a precise prediction towards the clinical severity of an LQTS1 variant cannot be made exclusively based on such in vitro studies.

## 2. Results

### 2.1. Case Descriptions

**Figure 1:**
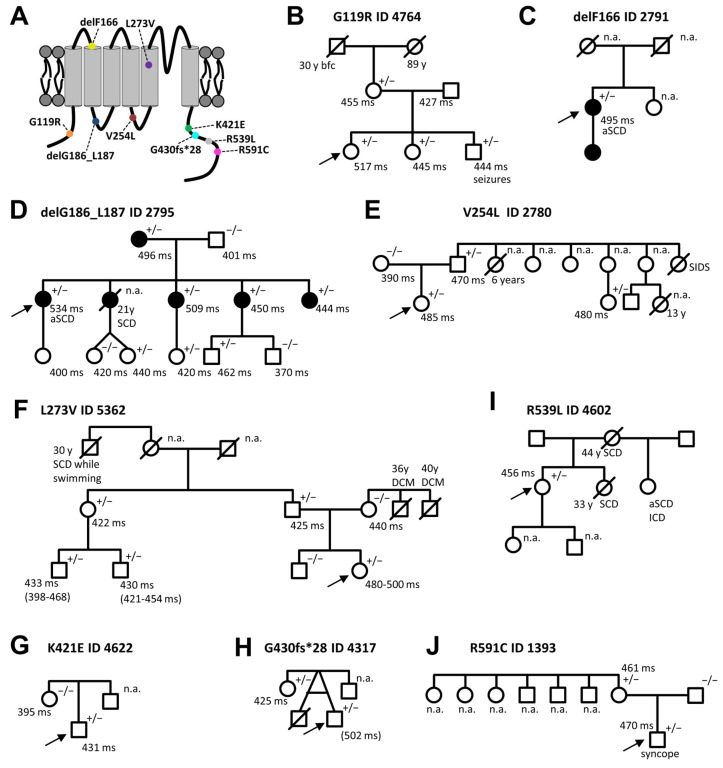
Localization of the identified *KCNQ1* variants in the KCNQ1 channel and pedigrees of the respective LQT1 index patients. (**A**) Topology of the KCNQ1 channel α-subunit with the localization of the variants highlighted by colored circles. (**B**) Pedigree of the index patient 4764, harboring the KCNQ1 variant G119R; (**C**) of the index patient 2791, harboring the KCNQ1 variant delF166; (**D**) of the index patient 2795, with the KCNQ1 variant delG186_L187; (**E**) of the index patient 2780, harboring the KCNQ1 variant V254L; (**F**) of the index patient 5362, with the KCNQ1 variant L273V; (**G**) of the index patient 4622, harboring the KCNQ1 variant K421E; (**H**) of the index patient 4317, with the KCNQ1 variant G430fs*28 (the QTc time of the index patient is provided in parenthesis, since a QTc prolongation occurred only once and temporarily); (**I**) of the index patient 4602 with the KCNQ1 variant R539L; and (**J**) of the index patient 1393, with the KCNQ1 variant R591C. Index patients are marked by arrows. Filled symbols indicate patients and family members with a previous diagnosis of LQTS with or without symptoms. Squares and circles represent male and female subjects, respectively. In the top right of the symbol, genetic information is given: +/−, heterozygous variant carrier and −/−, no variant carrier; not available (n.a.). Below the symbols, QTc time is and information about certain symptomatic or further diseases are given. SCD, sudden cardiac death; bfc, battlefield casualty; DCM, dilated cardiomyopathy; aSCA, aborted sudden cardiac death; SCD, sudden cardiac death; and SIDS, sudden infant death syndrome. Symbols with a line mark through them are deceased subjects, and the age (y) and cause of death are indicated.

This study functionally describes nine *KCNQ1* variants found in different index patients (IP) in a heterozygous state (Figure 1). The nine *KCNQ1* variants we studied are located in different segments of the α-subunit (Figure 1A). Different pathogenicity prediction tools, including PolyPhen2 [19], SIFT [20], SNAP [21], and PROVEAN [22], were applied to predict the pathogenic potential of all variants (Table 1).

**Figure 2:**
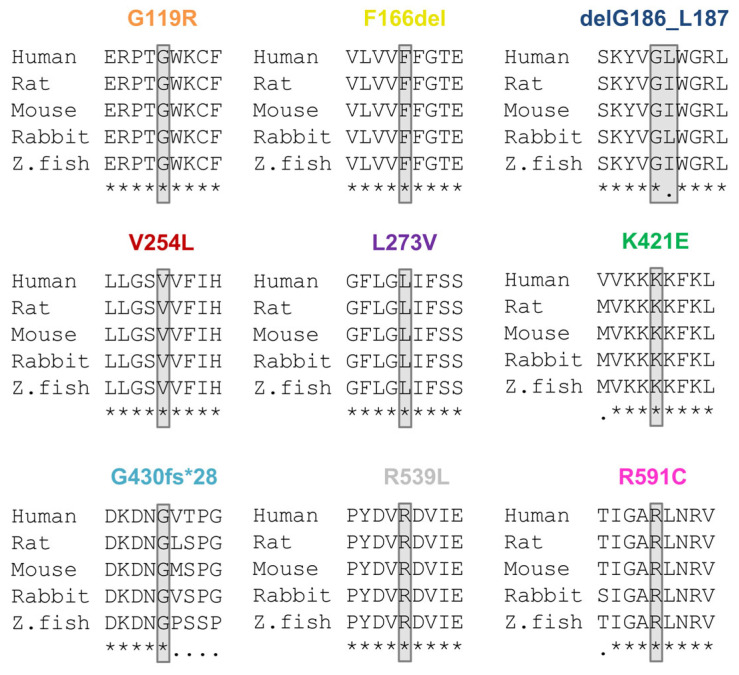
Protein sequence alignments illustrating the KCNQ1 variants in different orthologues. Partial amino acid alignments showing the mutated amino acids of the patients highlighted in gray. Below the sequences, conserved amino acids are marked with “*“and non-conserved amino acids are marked with “.”.

All amino acid positions mutated in the patients described here are highly conserved throughout orthologous channels (Figure 2).

The proband 4764 (G119R) (Figure 1B) harbours a single nucleotide exchange (missense mutation, non-truncating mutation) in a heterozygous manner at nucleotide 355 (c.355G>C), which leads to a glycine to arginine exchange in the channel protein, which is classified as a variant of uncertain significance. The variant is located in the cytosolic N-terminus (Figure 1A). The application of QT-prolonging drugs (citalopram and risperidone) caused a prolonged QTc interval (517 ms) in the ECG. Genetic testing of the mother, brother, and sister of the IP revealed that they also carried the G119R variants. Additionally, they present a borderline prolonged QT interval. The IP had no cardiac events, but suffered from depression. There was no family history for cardiac events like syncopes or SCD (Figure 1B).

The proband 2791 (delF166) (Figure 1C) has a deletion of 3 base pairs in the *KCNQ1* gene, that results in a loss of phenylalanine at codon 166. The female patient is a heterozygous carrier of the variant which is classified as likely pathogenic. This variant is located at the extracellular end of transmembrane segment S2 (Figure 1A). Patient 2791 suffered from syncopes and an aborted sudden cardiac arrest (aSCA) at the age of 61 years, which was her first cardiac event, reported to be caused by asystole. Therefore, a cardiac pacemaker was implanted. The QTc interval was prolonged to 495 ms. Trigger for cardiac events were usually physical exertion and physical activities of daily life, but events also occurred during sleep. Under beta-blocker therapy, the patient still suffered from repeated syncopes and another aSCA, with documented ventricular tachycardia which was the reason for ICD implantation. Regarding secondary disease, coronary heart disease requires a mention. The daughter anamnestically suffers from a LQTS, but her genomic DNA was however not available.

The proband 2795 (delG186_L187) (Figure 1D) has a 6 base pair deletion in the *KCNQ1* gene (c.556_561delGGCCTC). Therefore, the variant causes an in-frame deletion of the amino acids 186 and 187, not resulting in a frame shift and a premature stop codon. The variant is located in the S2–S3 linker (Figure 1A) and can be classified as pathogenic. The patient is heterozygous for the *KCNQ1* variant and suffered from syncopes and seizures since the age of 13 and survived a sudden cardiac arrest at the age of 31, at a family celebration (without beta-blocker) which resulted in severe brain damage. Triggers for cardiac events were exercise, contact with water, and activities of daily life. The female index patient showed a significantly prolonged QTc interval (QTc 534 ms) and a positive family history for SCD, in young family members. Besides the IP, all living family members are asymptomatic under beta-blocker therapy. One sister had died when she was 21 years old of sudden cardiac death (SCD) under unknown circumstances, without beta-blocker therapy.

The proband 2780 (V254L) (Figure 1E) has a heterozygous nucleotide exchange (missense mutation, non-truncating mutation) at nucleotide 760 (guanine to thymine), leading to a non-synonymous amino acid exchange of valine at codon 254 to leucine. The variant is located in the S4–S5 linker (Figure 1A) and can be classified as pathogenic. At the age of six, the female index patient suffered from a syncope for the first time. The cardiac events were triggered by swimming and trouble. In the ECG, a prolonged QT interval was documented (QTc 485 ms). There is a family history for sudden infant death syndrome (SIDS) (Figure 1E). Genetic testing of the father of IP 2780 was performed and the KCNQ1 V254L variant was detected as well. Consistently, the father also has a prolonged QTc interval (QTc 470 ms).

The proband 5362 (L273V) (Figure 1F) harbours a nucleotide exchange (missense mutation, non-truncating mutation) in the *KCNQ1* gene at nucleotide 819 (c.817C>G) which leads to a leucine to valine exchange at amino acid 273. The variant is located near the pore forming region in transmembrane segment S5 (Figure 1A) and is classified as likely pathogenic. Patient 5362 is heterozygous for the *KCNQ1* variant. In the first month of life, during a preventive medical examination, a heart murmur was noticed that led to a cardiologic check-up. The murmur was caused by an atrial septal defect. In the ECGs of the female index patient, prolonged QTc intervals (480–500 ms) were documented. There was a positive family history of cardiac events (Figure 1F). The father is an asymptomatic carrier of the variant and a paternal great-uncle had suffered from SCD, while swimming at the age of 30. In the family of the mother, two uncles of the IP died of SCD, probably caused by a known dilated cardiomyopathy (DCM). The family was instructed to also pay attention to the possible development of DCM.

The proband 4622 (K421E) (Figure 1G) harbours a single nucleotide exchange at position 1261 (adenine to guanine), leading to a lysine to glutamate exchange at amino acid position 421. The variant is located at the cytoplasmic C-terminus (Figure 1A) and is classified as a variant of uncertain significance. The IP 4622 suffered from non-epileptic seizures most likely due to reflex syncopes during infancy, as the recording of an implantable event recorder showed no heart rhythm disturbances during seizures, but QTc was prolonged. Under treatment with beta-blockers cardiac events were no longer reported. The mother of the IP carries no mutations/variants in LQTS associated genes and on the IP’s father’s side there is no information available (Figure 1G).

The proband 4602 (R539L) (Figure 1I) has a heterozygous single nucleotide exchange in the *KCNQ1* gene at nucleotide 1616 (guanine to thymine), leading to an arginine to leucine exchange at amino acid position 539. The variant is located in the cytoplasmic C-terminus (Figure 1A) and can be classified as likely pathogenic. The variant was found during genetic screening because of the positive family history (Figure 1I) and a borderline prolonged QTc interval (456 ms). The patient is asymptomatic, but the mother died at the age of 44 of SCD and the sister at the age of 33 of SCD. Another sister had an aSCA and received an implantable cardioverter-defibrillator (ICD).

The proband 4317 (G430fs*28) (Figure 1H) harbours a heterozygous deletion of 12 base pairs (c.1290_ 1301delGGTGACTCCTGG) in the *KCNQ1* gene, encoding for the amino acid 430–434, ultimately leading to a frame shift and a premature stop codon 28 amino acids downstream. The variant is located at the cytoplasmic C-terminus (Figure 1A) and is classified as a variant of uncertain significance. At the age of three, the patient suffered from a febrile convulsion. Under antibiotic treatment with cefotaxime and vancomycin, a prolonged QT interval was documented (QTc 502 ms) and therefore genetic screening of LQTS-associated genes revealed the KCNQ1 G430fs*28 variant. A subsequent genetic screening of the mother of the IP revealed the same variant, while she presents a normal QTc. The mother lost the twin of the IP due to intrauterine death, presumably resulting from a thrombocytopenia. There is no family history of syncopes or SCD.

The proband 1393 (R591C) (Figure 1J) harbours a heterozygous single nucleotide exchange in the *KCNQ1* gene at nucleotide 1771 (cytosine to thymine), leading to an arginine to leucine exchange at amino acid position 591, which is classified as pathogenic. The variant is located in the cytoplasmic C-terminus (Figure 1A). The first cardiac event of IP 1393 was a convulsive syncope at the age of five. A prolonged QTc interval (470 ms) was observed in the surface ECG recordings of the index patient. There was, however, no family history of cardiac events. The mother of the IP harbours the same *KCNQ1* variant and a prolonged QTc interval (461 ms) was observed in an ECG, even though the mother was asymptomatic. The siblings of the mother refused any genetic testing and clinical information was not available.

### 2.2. Electrophysiological Characterization of KCNQ1 Variants

**Figure 3:**
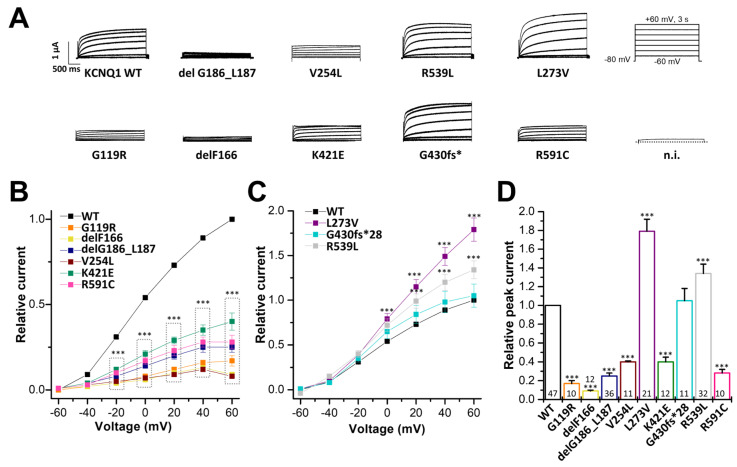
Electrophysiological properties of nine *KCNQ1* variants. (**A**) Representative current traces of wild-type KCNQ1 and the KCNQ1 variants after heterologous expression in *Xenopus laevis* oocytes (14.5 ng cRNA/oocyte). Voltage was clamped to potentials ranging from −60 to +60 mV in 20 mV increments, with steps of 3 s durations (the voltage protocol is illustrated on the right). n.i., current trace of non-injected oocytes at a voltage step from −80 to +60 mV. (**B**) Current-voltage relationships of variants with significantly reduced current amplitudes compared to wild-type KCNQ1. Current-voltage relationships (I/V curves) were obtained by analyzing the current amplitude at the end of each voltage step, normalized to the current of wild-type KCNQ1 channels at +60 mV. Note that all data were normalized to the currents recorded for the wild-type channels at +60 mV of the respective recording day. (**C**) Current-voltage relationships of variants with significantly increased current amplitudes and variants without changes in the current amplitudes. (**D**) Current amplitudes analyzed at +40 mV (normalized to the amplitudes of wild-type KCNQ1 (WT)). All the data, including that of the wild-type recordings, were divided by the average current amplitude of the wild-type at +40 mV of the respective recording day. The current amplitude of non-injected oocytes at +40 mV was about 150 nA. Numbers of oocytes recorded are indicated within the bar graphs. Values are expressed as means ±S.E.M. Error bars represent S.E.M. values. Significance was assessed using two-tailed Student’s *t*-test. Asterisks indicate significance: ***, *p* < 0.001.

For the variant-specific interpretation of changes in the APD and ECG, only the recordings of the heterozygous conditions (WT/variant KCNQ1 plus KCNE1) are in fact necessary. Yet, we recorded homozygous and heterozygous conditions with and without KCNE1. This approach was chosen, as we thought that it was of clinical interest in how *I*_Ks_ currents might be affected in homozygous patients, since such conditions might occur in other cases/families, or in the future. The measurements of KCNQ1 mutants in the homozygous and heterozygous state without KCNE1 may also be of putative future interest, as there are other tissues where KCNQ1 may not co-assemble with its ß-subunits, and thus this information might be valuable in the future, helping the interpretation of putative non-cardiac manifestations in LQTS. To examine the electrophysiological consequences of the KCNQ1 variants, we first expressed wild-type KCNQ1 or variants in the homozygous state in *Xenopus laevis* oocytes, and performed voltage-clamp recordings. The KCNQ1 variants G119R, delF166, delG186_L187, V254, K421E, and R591C conducted significantly less current than wild-type KCNQ1 channels (Figure 3A,B,D). Conversely, the KCNQ1 variants L273V and R359L led to significantly increased currents, compared to that of the wild-type KCNQ1 channels (Figure 3A,C,D). On the other hand, no significant differences in current amplitudes were observed for the G430fs*28 variant (Figure 3C,D).

### 2.3. Electrophysiological Characterization of KCNQ1 Variants Co-Expressed with KCNQ1 Wild-Type Subunits

**Figure 4:**
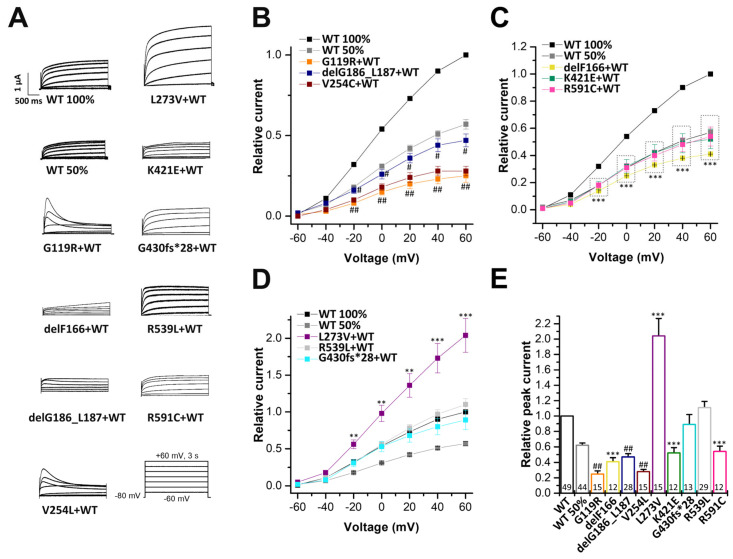
Electrophysiological characterization of the KCNQ1 variants co-expressed with wild-type KCNQ1 channels. (**A**) KCNQ1 wild-type (14.5 ng cRNA/oocyte, WT 100%), KCNQ1 wild-type (7.25 ng cRNA/oocyte, WT 50%) or KCNQ1 wild-type (7.25 ng cRNA), plus KCNQ1 mutant (7.25 ng/oocyte) were expressed in *Xenopus laevis* oocytes. In voltage-clamp recordings, voltage was stepped to potentials between −60 and +60 mv in 20 mV-steps lasting 3 s, starting from a holding potential of −80 mV (voltage protocol illustrated at the bottom). Representative current traces for wild-type KCNQ1 (WT 100%), KCNQ1 WT 50% or KCNQ1 WT co-expressed with the indicated variants are shown. (**B**) Current-voltage relationships obtained by plotting the current at the end of each voltage step, normalized to the current of KCNQ1 wild-type 100%. In order to obtain the current-voltage relationship (I/V curve), all wild-type recordings were normalized to the value at +60 mV. The data of all the other constructs were also divided by the average current amplitude of the wild-type, at +60 mV of the respective recording day. Illustrated are the dominant-negative variants with significantly reduced current amplitudes compared to KCNQ1 wild-type 50% (marked with # or ##), (**C**) KCNQ1 variants with significantly reduced current amplitudes compared to KCNQ1 WT 100% (marked with ***), and (**D**) KCNQ1 variants with unchanged current amplitudes or even significantly increased current amplitudes compared to KCNQ1 WT 100% (marked with ** or ***). (**E**) Current amplitudes analyzed at +40 mV and normalized to the currents of wild-type KCNQ1 channels. All the data, including that of the wild-type recordings, were divided by the average current amplitude of the wild-type at +40 mV of the respective recording day. Numbers of oocytes recorded are indicated within the bar graphs. Values are expressed as means ± S.E.M.. Error bars represent S.E.M. values. Significance was assessed using two-tailed Student’s *t*-test. Asterisks indicate significance compared to wild-type KCNQ1 100%: **, *p* < 0.01; ***, *p* < 0.001 or to KCNQ1 wild-type 50%: #, *p* < 0.05 or ##, and *p* < 0.01.

To gain insights into the electrophysiological behaviour of the variants in the heterozygous state, the variants were co-expressed with wild-type KCNQ1, by injecting similar amounts of wild-type and mutant cRNA. In addition, we expressed 50% of wild-type KCNQ1, mimicking a haploinsufficiency. To this end, we injected half the amount of the wild-type KCNQ1 cRNA. In these experiments G119R, delG186_L187, and V254L showed significantly reduced current amplitudes, compared to that of oocytes injected with half the amount of wild-type KCNQ1 cRNA. Thus, these variants must have a dominant-negative effect on wild-type KCNQ1 channels (Figure 4A,B,E). The delF166, K421E, and R591C variants did not present a dominant-negative effect, but showed significantly reduced current amplitudes when co-expressed in complex with wild-type KCNQ1 channels (Figure 4C,E). In contrast, G430fs*28 and R539L showed no significant current reduction (Figure 4A,D,E), and L273V, expressed together with wild-type KCNQ1, even showed significantly increased current amplitudes (Figure 4A,D,E).

### 2.4. Electrophysiological Characterization of Homomeric KCNQ1 Variants Co-Expressed with KCNE1

**Figure 5:**
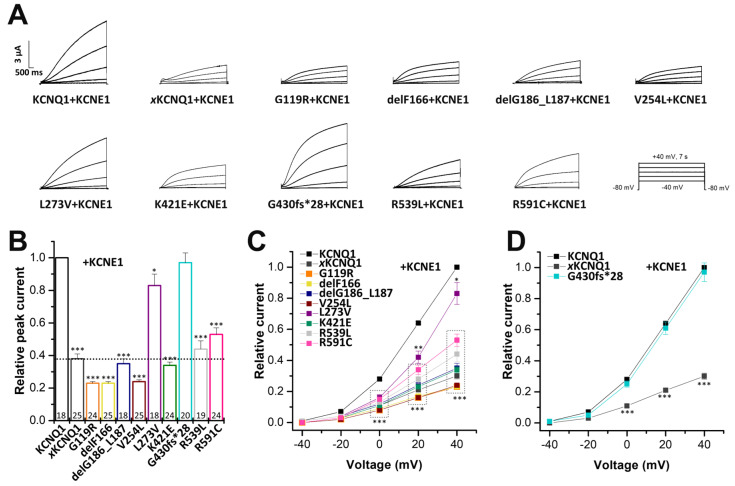
Electrophysiological properties of KCNQ1 variants co-expressed with KCNE1. (**A**) *Xenopus laevis* oocytes were injected with 81.2 ng wild-type KCNQ1 cRNA or the indicated mutant KCNQ1 cRNA, and all constructs were co-injected with 1 ng of KCNE1 cRNA. In addition, some oocytes were injected with 1 ng KCNE1 alone to quantify the amplitude of the endogenous *xI*_Ks_. Voltage was stepped to potentials ranging from −40 to +40 mv in +20 mV steps lasting for 7 s, each starting from a holding potential of −80 mV. The voltage protocol is indicated on the right bottom corner. Representative current traces for wild-type KCNQ1 plus KCNE1, *x*KCNQ1 plus KCNE1 or the indicated mutants plus KCNE1 are shown. (**B**) Current amplitudes analyzed at +40 mV and normalized to wild-type KCNQ1 plus KCNE1. All the data, including that of the wild-type recordings, were divided by the average current amplitude of the wild-type at +40 mV of the respective recording day. Numbers of oocytes are indicated within the bar graph. (**C**) Current-voltage relationships obtained by plotting the current at the end of each voltage step for each voltage applied normalized to wild-type KCNQ1 plus KCNE1. In order to obtain the current-voltage relationship (I/V curve), all wild-type recordings were normalized to the value at +40 mV. The data of all the other constructs were also divided by the average current amplitude of the wild-type at +40 mV of the respective recording day. KCNQ1 variants with a significant current reduction are illustrated in (**C**) and a variant with no significant effect in (**D**). Values are expressed as means ± S.E.M. Error bars represent S.E.M. values. Significance was assessed using two-tailed Student’s *t*-test. Asterisks indicate significance: *, *p* < 0.05; **, *p* < 0.01; ***, and *p* < 0.001.

As described above, KCNQ1 co-assembles in the heart with its subunit KCNE1 to form the *I*_Ks_. Therefore, we co-expressed the KCNQ1 variants with KCNE1, to record *I*_Ks_ (Figure 5). We also injected KCNE1 alone, to quantify the current amplitudes of the endogenous *Xenopus I*_Ks_ (*xI*_Ks_), formed by endogenous *Xenopus* KCNQ1 subunits and the heterologously expressed KCNE1. The G119R, delF166, delG186_L187, V254L, K421E, R539L and R591C variants co-expressed with KCNE1 presented a significant *I*_Ks_ reduction, with current amplitudes in the range of endogenous *xI*_Ks_ recorded by the injection of KCNE1 alone (Figure 5A-C). The homomeric G119R, delF166, and V254L variants co-expressed with KCNE1 presented even a dominant-negative effect on the endogenous xKCNQ1, as the resulting *I*_Ks_ amplitudes were even smaller than for the endogenous *xI*_Ks_, recorded by the injection of KCNE1 alone (Figure 5B-C). The L273V co-expressed with KCNE1 also showed significantly reduced currents compared to KCNQ1 wild-type co-expressed with KCNE1, albeit the strength of the effects was dependent on the voltage at which the analyses was performed. In contrast, G430fs*28 co-expressed with KCNE1 did not significantly change the current amplitudes, compared to that of wild-type KCNQ1 co-expressed with KCNE1 (Figure 5B,D).

### 2.5. Electrophysiological Characterization of KCNQ1 Variants Co-Expressed with Wild-Type KCNQ1 and KCNE1

**Figure 6:**
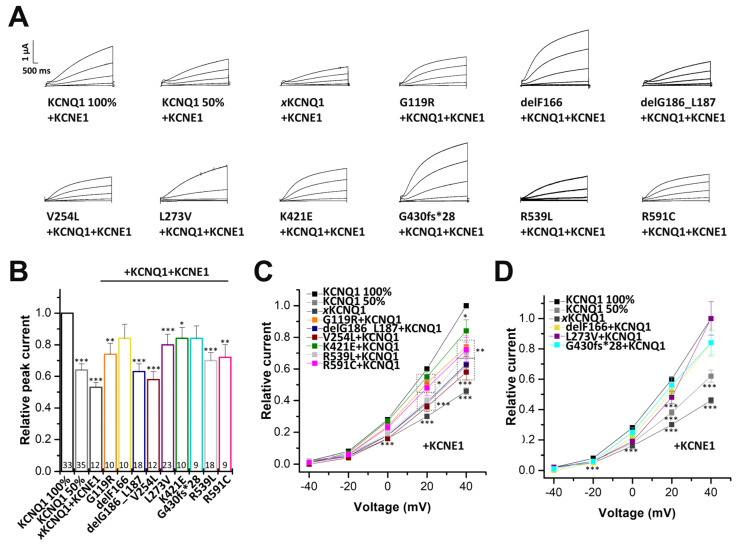
Electrophysiological properties of KCNQ1 variants co-expressed with wild-type KCNQ1 and KCNE1. (**A**) *Xenopus laevis* oocytes were injected with different controls. KCNQ1 100% = 81.2 ng wild-type cRNA, plus 1 ng KCNE1; KCNQ1 50% = 40.6 ng wild-type KCNQ1 cRNA and 1 ng KCNE1. The heterozygous KCNQ1 variants were studied by injecting 40.6 ng cRNA of the respective variants plus 40.6 ng wild-type KCNQ1 cRNA, together with 1 ng of KCNE1 cRNA. In addition, oocytes were injected with 1 ng of KCNE1 alone, to quantify the endogenous *xI*_Ks_ formed by co-assembly of KCNE1 with endogenous *x*KCNQ1. Voltage was stepped for 7s to potentials ranging from −40 to +40 mV in +20 mV increments. The holding potential was −80 mV. Representative current traces of the indicated constructs are shown. (**B**) Current amplitudes were analyzed at +40 mV and normalized to 100% KCNQ1 wild-type, plus KCNE1. All the data, including that of wild-type recordings, were divided by the average current amplitude of the wild-type at +40 mV of the respective recording day. Numbers of oocytes are indicated within the bar graph. Currents of the L273V variant were analyzed at +20 mV, as the effects of this variant were voltage-dependent, but clearly present in the physiological range. (**C**) Heterozygous variants with significantly reduced current amplitudes compared to KCNQ1 WT 100% + KCNE1. (**D**) Heterozygous variants with no significant changes in current amplitudes. Note that for the L273V variant studied in the heterozygous state, in the presence of KCNE1, significant current reductions are observed at membrane potentials lower than +40 mV. Values are expressed as means ± S.E.M.. Error bars represent S.E.M. values. Significance was assessed using two tailed Student’s *t*-test. Asterisks indicate significance: *, *p* < 0.05; **, *p* < 0.01; ***, and *p* < 0.001.

Next, we examined the effect of the KCNQ1 variants co-expressed with equal amounts of wild-type KCNQ1, in the presence of KCNE1 (mimicking the heterozygous state of the patients in the heart). The G119R, delG186_L187, V254L, K421E, R539L and R591C variants significantly reduced current amplitudes compared to wild-type *I*_Ks_ (Figure 6A–C). Here, delG186_L187, V254L and R539L showed the strongest current reduction (*p* < 0.001). The heteromeric L273V variant co-expressed with KCNE1 also showed significantly reduced currents compared to wild-type KCNQ1, co-expressed with KCNE1. Similarly, as described above, for the homomeric variant in the presence of KCNE1, the strength of the effects were dependent on the voltage at which the analyses was performed (Figure 6B,D). In contrast, we observed no notable change compared to *I*_Ks_ wild-type, for the variants delF166 and G430fs*28 (Figure 6A,B,D).

### 2.6. The delG186_L187, L273V and R539L Variants Alter the Voltage-Dependent Gating of the Heterozygous I_Ks_ Channel Complexes

**Figure 7:**
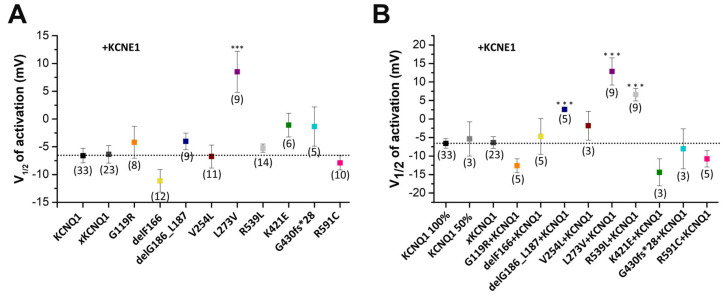
Voltage-dependence of activation of the KCNQ1 variants expressed in a homozygous or heterozygous complex with KCNE1. (**A**) Voltage-dependence of activation for homozygous KCNQ1 variants co-expressed with KCNE1 (81.2 ng KCNQ1 variants plus 1 ng KCNE1 or (**B**) in a heterozygous state with wild-type KCNQ1 and KCNE1 (40.6 ng KCNQ1 + 40.6 ng KCNQ1 variants + 1 ng KCNE1. Controls were: KCNQ1 100% = 81.2 ng KCNQ1 + 1 ng KCNE1, KCNQ1 50% = 40.6 ng KCNQ1 + 1 ng KCNE1, *x*KCNQ1 = 1 ng KCNE1. The tail current of each variant after the 7 s pulse was normalized to their maximum current. Recordings were performed with the protocol as described in Figure 6. The tail currents recorded after the 7 s pulse were normalized to the respective maximal tail current of each recording, to obtain the conductance/voltage (G/V) curves. Normalized tail currents were fitted to the Boltzmann function. The voltage of half-maximal activation (V_1/2_) of the respective construct is illustrated, together with the numbers of oocytes analyzed. Values are expressed as means ± S.E.M. Error bars represent S.E.M. values. Significance was assessed using two tailed Student’s *t*-test. Asterisks indicate significance: ***, and *p* < 0.001.

In the homozygous state of the KCNQ1 variants co-expressed with KCNE1, only L273V presented a significant rightward shift in the voltage of half-maximal activation (V_1/2_) (Figure 7A). Next, we analyzed whether the heterozygous KCNQ1 variants, in complex with KCNE1, affect the voltage-dependence of activation of *I*_Ks_ channel complexes containing KCNE1 (Figure 7B and Figure S1). Strikingly, in the heterozygous state, relevant for the patients, even three more variants (delG186_L187, L273V and R539L) showed a rightward shift in the V_1/2_ of activation (Figure 7B).

### 2.7. In Silico Modelling of the Changes to the Human Ventricular Action Potentials for the Different KCNQ1 Variants

**Figure 8:**
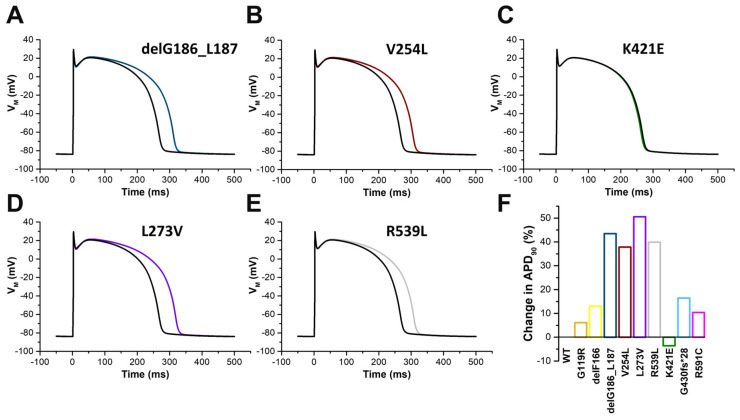
In silico modelling of the human ventricular action potential (AP) using the ten Tusscher modelling of human ventricular myocytes for the different KCNQ1 variants. Illustrated are the calculated Aps for control (black) and (**A**) delG186 (blue), (**B**) V254L (red), (**C**) K421E (green), (**D**) L273V (blue), and (**E**) R539L (gray). (**F**) Changes in the calculated APD_90_ compared to wild-type are plotted for the different KCNQ1 variants.

Next, we performed in silico modelling of the human ventricular action potential (AP) using the ten Tusscher modelling of human ventricular myocytes (Material and Methods, Figure 8). To this end, we integrated the changes to the current amplitudes and the V_1/2_ of the different variants in the heterozygous complex with KCNQ1 and KCNE1 into the model. AP modelling revealed prolonged action potentials for all variants examined (Figure 8), with K421E as the exception (Figure 8C,F). The most prominent AP prolongations were observed for delG186_L187 (Figure 8A,F), V254L (Figure 8B,F), L273V (Figure 8D,F), and R539L (Figure 8E,F) variants, while a minor AP prolongation of less than ten percent was observed for the G119R variant (Figure 8F).

**Figure 9:**
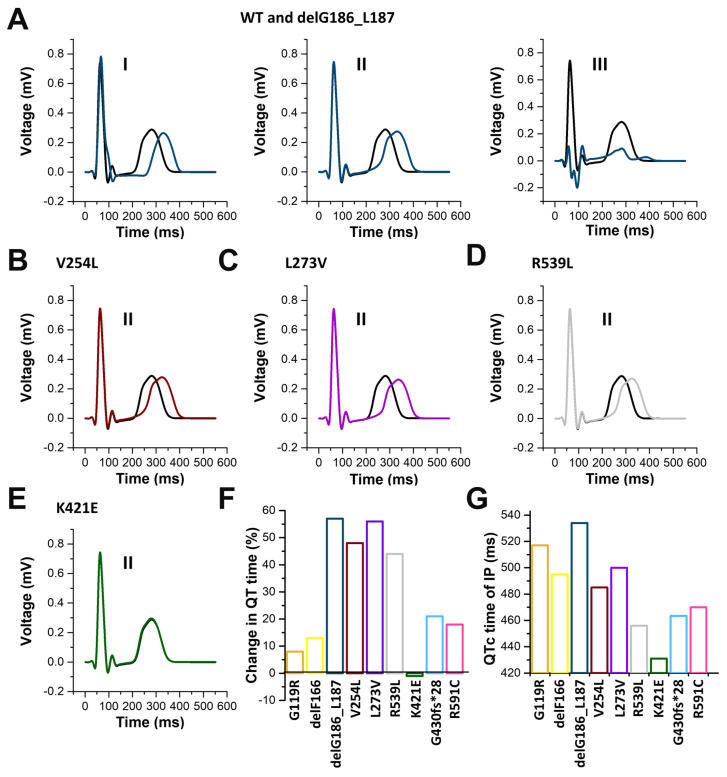
Simulated surface electrocardiograms (ECGs) based on the variant-specific changes to the action potentials predicted by the *ten Tusscher* model of human ventricular myocytes (Figure 8). (**A**) ECG of leads I, II, and III of wild-type KCNQ1 (black) and the variant delG186_L187 (blue). Examples of lead II of the calculated surface ECG for wild-type (black) and the variants (**B**) V254L (brown), (**C**) L273V (purple), (**D**) R539L (gray), and (**E**) K421E (green). (**F**) Quantification of the percentage of change in the QT time for the different variants. (**G**) QTc times of the index patients with the different KCNQ1 variants. Note, that for the G430fs*28 variant, the mean QTc time of the index patient and the heterozygous mother was illustrated, since the QTc prolongation of the index patient occurred only once and temporarily.

To compare these findings with the severity of the QT changes observed in the index patients, we performed in silico modelling experiments of surface electrocardiographs (ECGs), implementing the predicted changes determined by the ten Tusscher model of human ventricular myocytes. As an example, Figure S2 illustrates the computationally determined ECG for all 12-leads with wild-type controls and the KCNQ1 del186_187 variant. Prolonged QT times were calculated for all KCNQ1 variants analyzed in our study (Figure 9). The most pronounced QT time prolongation were observed for delG186_L187 (Figure 9A,G), V254L (Figure 9B,G), L273V (Figure 9C,G), and R539L (Figure 9D,G) variants. Similar to the AP modelling, the QT prolongation was minor for the G119R variant (QTc change < 10% compared to wild-type), while K421E had no effect on the calculated QT time (Figure 9E,F). The in silico calculated QT time prolongation of the nine different variants roughly correlated with the variant-specific QTc times of the affected family members (Figure 9F,G), with the exceptions of G119R and delF166.

## 3. Discussion

In the present study, we first describe and functionally characterize seven novel KCNQ1 variants, namely G119R, delF166, delG186_L187, L273V, K421E, G430fs*28, and R539L. The two other variants (V254L and R591C) that were also characterized in the current study were initially reported in a genetic screening of LQTS patients by Napolitano et al. [7].

The carrier of the variant of uncertain significance G119R (IP 4764) presented a strongly prolonged QTc interval during a febrile infection treated with QT prolonging drugs. According to our electrophysiological results of the heterologous expression of the heterozygous *I*_Ks_ channel complex with the variant, a current reduction of 26% is expected, while the variant does not show a dominant-negative effect. Accordingly, only a mild phenotype is expected. The IP’s mother and brother present no symptoms and have just borderline prolonged QT intervals underlining the mild phenotype, but it should be highly recommended to avoid any QT prolonging drugs. If the seizures of the brother of the IP are associated with cardiac arrhythmia due to the reduced *I*_Ks_ remains unclear, as no further information concerning the neurological examinations or the trigger for the seizures is available. Although recent Clinvar entries classify the exchange as a variant of unknown significance (VUS), based on our findings, this variant should at least be judged as a functional risk allele which can lead to serious QTc prolongation under certain circumstances (e.g., QT prolonging drugs).

The proband 2791 with the likely pathogenic variant delF166 is severely affected by syncopes and aSCA, despite beta-blocker therapy. The KCNQ1 variant showed a total loss-of-function and the KCNQ1 channel variant co-expressed with wild-type channels a mildly dominant-negative effect. But surprisingly the heteromeric delF166 variant in co-expression with wild-type KCNQ1 and KCNE1 showed only a minor *I*_Ks_ reduction. According to our electrophysiological data and ECG modelling, the heterozygous variant carrier should only have a very mild phenotype or no symptoms. Thus, a further examination on any other causes explaining the cardiac events should be considered, i.e., a digenic mutational state. Also the coexistence of different diseases manifesting through SCD and syncopes might be possible [23].

The IP 2795 with the pathogenic variant delG186_L187 has a severe phenotype with aSCA prior to beta-blocker therapy at the age of 31 years, which resulted in severe brain damage and syncopes since she was 13-year-old. The voltage-clamp recordings predict a strong current reduction of the heterozygous *I*_Ks_ channel complex. In addition, to this loss-of-function in the current amplitude, the voltage-dependence of activation showed a rightward shift in the heterozygous state with KCNQ and KCNE1. Interestingly, the deletion of the two amino acids (glycine 186 and leucine 187) causes a repositioning of positively charged residues in the S2–S3 linker (for example the arginine residues at amino acid positions 190, 192, and 195), which could add to the altered voltage-dependence of the variant, i.e., by a dysfunctional PIP_2_ binding [24]. 

The proband 2780 with the pathogenic variant V254L had her first syncope at the age of six years, before beta-blocker therapy was started. Note, that we provide the first functional characterization of this variant, which was previously mentioned by Napolitano et al. [7]. Notably, in our family there was a marked positive history for sudden cardiac death in young family members. In our dataset the KCNQ1 variant caused the most pronounced loss-of-function in the heterologously expressed heterozygous *I*_Ks_ channel complex with wild-type KCNQ1 and KCNE1. Thus, the voltage-clamp study is in agreement with the severe manifestation of the LQTS in the index patient.

The proband 5362 (L273V) presented without symptoms, despite the QTc prolongation. However, there was a paternal great-uncle that died while swimming at 30-years-old and two maternal uncles who died of sudden cardiac death (SCD) because of dilated cardiomyopathy (DCM). Strikingly, the homomeric KCNQ1 channel variant and the heterozygous complex of the variant co-expressed with wild-type KCNQ1 channels, both showed a gain-of-function. These observations are in agreement with a study by Seebohm et al. who reported that amino acids glycine 272, leucine 273, and valine 307 are important for the inactivation of the KCNQ1 channel, and that introducing a valine at position 273 leads to a complete loss of inactivation [18]. However, residues at position 273 also determine the efficiency of KCNE1 to alter the gating of KCNQ1 channels [25]. In our study, this critical role of the residue at this position for the KCNQ1 modulation by KCNE1 became very evident. Here, after co-expression with KCNE1 the effects were inverted, which resulted in a significant loss-of-function. This loss-of-function in the heterologously expressed *I*_Ks_ channel complex containing the L273V variant, is primarily caused by a rightward shift of the voltage-dependence of activation.

In the current study, we provide the first functional characterization of the KCNQ1 K421E variant which is rated as VUS. The proband 4622 (K421E) presented with recurrent non-epileptic seizures and as a QT prolongation was observed during the seizures, a genetic testing of LQTS-associated genes was performed. However, subsequently no QTc prolongation was observed in further ECGs, recorded under resting conditions. The KCNQ1 channel variant causes a loss-of-function. In the heterologously expressed heterozygous *I*_Ks_ channel complex with KCNQ1 and KCNE1, we observed a significant current reduction of 16%. While according to our voltage-clamp studies, patients carrying the variant in a heterozygous manner are expected to have a less pronounced phenotype, homozygous variant carriers are expected to have a severe phenotype, as we observed here a strong current reduction of 66%. As the index patient was a heterozygous carrier of the variant other causes of recurrent syncopes should be considered.

The proband 4317 (G430fs*28, VUS) experienced a febrile convulsion at the age of three years and had a documented prolonged QT interval under antibiotic therapy with cefotaxime and vancomycin. We found no significant electrophysiological evidence for the pathogenicity of the G430fs*28 variant. The G430fs*28 variant caused a relatively small reduction of the *I*_Ks_ channel complex with KCNE1, independent of the variant being expressed homomeric with KCNE1, or in a heterozygous mixture with wild-type KCNQ1 plus KCNE1. Although the magnitude of current reduction in the heterologously expressed heterozygous *I*_Ks_ channel complex was similar to that of K421E and delF166, the changes induced by this variant did not reach statistical significance. Consistently, the IP and his mother which are carrying the variant G430fs*28 showed normal QTc intervals in a follow-up visit.

The proband 4602 with the likely pathogenic variant R539L has a highly positive family history for sudden cardiac death. The KCNQ1 channel variant, in the absence of KCNE1, shows a gain-of-function, similar to the L273V variant. After assembly with KCNE1, we found a loss-of-function that is agreement with the observed LQTS. The variant is located at a highly conserved region of the C-terminus (amino acids 509–575), which is an important factor determining the relevance of C-terminal KCNQ1 variants [26]. Notably, the C-terminal residues R539 and R555, together with the S4-S5 linker residue R243, were reported as binding sites for PIP_2_. As a result, of the substitution of the positively charged arginine at position 539 to an uncharged leucine, the PIP_2_ sensitivity of this KCNQ1 variant might be decreased, similar to a previously studied R539W mutation [27].

The R591C was previously reported by Napolitano [7]. Although this variant is categorized as pathogenetic, the consequences of the amino acid exchange were never functionally studied. The index patient 1393 carrying this variant had a convulsive syncope at the age of five and a prolonged QTc interval. In the heterologously expressed heterozygous *I*_Ks_ channel complex with KCNQ1 and KCNE1, we observed a significant current reduction of 28%. Thus, our functional data is in agreement with the observed QTc time prolongation in our patients and the previous categorization as pathogenetic LQT1 variant.

We aimed at correlating the QTc time of the index patients with the changes in QT times from simulated ECGs, which were based on the variant-specific changes to the action potentials predicted by the *ten Tusscher* model of human ventricular myocytes. However, there are some systematic limitations to our approach, as only QTc times of single index patients or family members were available, and for correlation, a multitude of systematically recorded ECGs from affected and non-affected family members of different large families should be compared. In addition, ECGs of the index patients were not obtained in a systematic manner and patients were treated with medications in a different manner, i.e., some patients were treated with beta-blockers and other variant carriers not. In addition, the serum potassium levels were not available at the time of the ECG recordings for all patients. There are some further limitations to our modelling, as we did not consider activation and deactivation kinetics of the variants, as these parameters may also affect the rate of AP repolarization. Thus, the ECG modelling is rather an estimation, as more parameters than the AP parameters need to be implemented to gain more precise results. Nevertheless, the ECG modelling data already provides a valuable first estimation of the effects caused by the variants. In addition, there are also limitations to the action potential and surface ECG modelling. For instance, the *I*_Ks_ plays a comparatively big role in the *ten Tusscher* model, compared to some other models. The functional significance of the slow versus rapid component of the delayed rectifier K^+^ current (*I*_Ks_ versus *I*_Kr_), in humans and other species, also remains a matter of investigation [28]. Thus, our correlation attempts are limited in the predictive value and should be interpreted with caution.

Noteworthy, the heterologous *Xenopus laevis* expression system which we have used here does not reflect the perfect physiological conditions, as lower temperatures compared to that in humans are used, which may influence protein stability and trafficking, while the AP and ECG modelling were performed at 37 °C. Yet, we used the oocyte expression system, as it provides several very important advantages. For example, it is possible to express precise amounts and ratios of the channels and subunits in each cell/oocyte. In contrast, transfected cells will uptake a variable amount of cDNA, with a 1:1 ratio, mimicking heterozygosity, not guaranteed. Similarly, there will be no fixed channel to KCNE1 stoichiometry in every cell. In the oocyte system on the other hand, every cell has as a precise amount of injected cRNA for all constructs, and in addition, there are no problems from variations in the series resistance arises. Thus, with voltage-clamp experiments in oocytes, it is possible to detect minor changes in current amplitudes, which is relatively difficult or not possible in patch clamp recordings of mammalian cells. However, despite the methodological limitations, we think that the study sufficiently describes or suggests how the novel LQT1 variants might act in vivo, being well aware that an ultimate proof of a disease-causing mechanism is very hard to achieve.

Nevertheless, we found that the electrophysiological data of the variants correlates with the severity of the clinically diagnosed phenotype in most cases, however, not in all. We conclude that electrophysiological studies combined with in silico modelling approaches are valuable components for the interpretation of KCNQ1 variants, but assessing the severity of a variant demands the consideration of other clinical factors. Our study widens the spectrum of novel disease-associated KCNQ1 variants, providing an important or necessary step towards a sound interpretation of the pathogenicity of variants identified in Romano-Ward Syndrome.

## 4. Materials and Methods

### 4.1. Clinical Evaluation

Between 1999 and 2007, KCNQ1 variants were discovered in genomic DNA of Index patients (IP) described in this study. The diagnosis of LQTS was set by a cardiologist specialized on inherited arrhythmia syndromes at the Hospital of the Ludwig Maximilian University of Munich. Detailed medical history focusing on LQTS relevant comorbidities, the family history for 3 generations (if possible), and 12-lead resting ECGs were taken. According to Goldenberg et al. [29] we considered a QT interval corrected for the heart rate using Bazett’s formula (QTc) ≤ 450 ms in men and ≤ 460 ms in women to be normal. Informed consent prior to genetic investigations was collected for all patients in this study, complying with the ethical standards of the 1964 Declaration of Helsinki and its latest revision.

### 4.2. Classification of Variants

The consensus guidelines of the American College of Medical Genetics (ACMG) [30] were applied to classify variants as pathogenic (P), likely pathogenic (LP), or as a variant of unknown significance (VUS). This classification included evidence from common databases (Genome Aggregation Database [31], 1000 Genomes Project [32], NCBI ClinVar [33]), prior reports in the literature, functional evidence, the presence of cosegregation data, and the application of in silico prediction tools (PROVEAN [22], PolyPhen-2 [19], SIFT [34], and SNAP2 [35]).

### 4.3. Molecular Biology

Genomic DNA was isolated from blood samples of the IP. The exons and intronic splice sites of KCNQ1 were PCR amplified and sequenced. DNA sequence information was compared with the KCNQ1 wild-type sequence (NM_000218.3). Human KCNQ1 was cloned into the pSP64T vector and Quik-Change Site-Directed Mutagenesis Kit (Agilent Technologies, Santa Clara, CA, USA) was used to introduce the variants. cDNA was linearized with EcoRI (Fermentas, St. Leon-Rot, Germany) and cRNA synthesized in vitro using the mMESSAGEmMACHINE^®^SP6 kit (Ambion, Austin, TX, USA). cRNA concentration was quantified using a spectrophotometer (Genequant, Pharmacia Biotech, Piscataway, NJ, USA) and quality was checked by agarose gel electrophoresis.

### 4.4. Electrophysiology

Oocytes were taken from ovarian lobes of anesthetized *Xenopus laevis* toads, anaesthetized with 2 g/l tricaine-methansulfonate (SIGMA, Taufkirchen, Germany). To remove residual connective tissue, oocytes were subsequently treated with collagenase (2 mg/mL Worthington type II, (Nordmark, Uetersen, Germany)) in OR2 solution (NaCl 82.5 mM, KCl _2_ mM, MgCl_2_ 1 mM, HEPES 5 mM, pH 7.4; all from SIGMA, Taufkirchen, Germany), for 120 min. Afterwards oocytes were stored at 18°C in ND96 recording solution (NaCl 96 mM, KCl_2_ mM, CaCl_2_ 1.8 mM, MgCl_2_ 1 mM, HEPES 5 mM, pH 7.5), supplemented with Na-pyruvate (275 mg/l), theophylline (90 mg/l), and gentamicin (50 mg/l) (all from SIGMA, Taufkirchen, Germany). Oocytes were injected with 50.6 nl cRNA. For the co-expression experiments of KCNQ1 with KCNE1, more KCNQ1 cRNA (81.2 ng per oocyte) was injected, compared to our experiments expressing KCNQ1 alone (14.5 ng/oocyte). This adaptation was carried out, as we experienced that more KCNQ1 cRNA was necessary to yield an *I*_Ks_ expression of about several µA. Standard two electrode voltage-clamp experiments were performed at room temperature (21–22 °C) with an Axoclamp 900A amplifier, a Digidata 1440A and pClamp10 software (Axon instruments, San Jose, CA, USA), 72 h after cRNA injection. The microelectrodes were made from glass pipettes (Science products, Hofheim, Germany) pulled with a DMZ-Universal puller (Zeitz, Martinsried, Germany) to a resistance of 0.2–1.0 MΩ, when filled with 3 M KCl (SIGMA, Taufkirchen, Germany). Currents were induced according to different voltage protocols: 3 s pulses were applied, in 20 mV steps from −60 mV to +60 mV, to oocytes expressing KCNQ1 channels and 7 s pulses were applied, in 20 mV steps from −40 mV to +40 mV, to oocytes expressing the *I*_Ks_-channels.

### 4.5. In Silico Model of Human Ventricular Myocytes

To underpin the experimental findings mechanistically and bridge biological levels of integration, we conducted in silico experiments. A temperature of 37 °C was applied in all models. Cardiomyocyte membrane dynamics were represented by the ten Tusscher et al. 2006 model of human ventricular myocytes [36]. *I*_Ks_ was controlled by an activation and an inactivation gate, with the same half (in)activation voltage V_1/2_ in this model. For each variant, V_1/2_ was shifted according to the difference in control in the wet lab experiments. We performed two series of simulations: one with only V_1/2_ changes being considered and the second with also the maximum conductance gKs being affected.

On a single cell level, the myocytes were paced to limit cycle at 2 Hz before evaluating the transmembrane voltage and *I*_Ks_ course during an action potential, as well an APD90. The experiments were run with the open cardiac electrophysiology simulator openCARP [37], using the default numerical settings. The simulation setup is publicly available “https://dx.doi.org/10.35097/580 (accessed on 29 March 2022)”. 

On tissue scale, we computed the spatio-temporal distribution of the transmembrane voltage on the mean geometry of a biventricular statistical shape model [38,39], using the monodomain model, and obtained the 12-lead electrocardiogram by solving the forward problem of electrocardiography with the boundary element method in a homogeneous, anatomically accurate torso model. Size, position, and delay of three initially activated regions located on a fast-conducting subendocardial layer, representing the fascicles of the His-Purkinje system, were optimized to obtain a physiological QRS complex. Moreover, gKs gradients were adjusted along the apico-basal, transmural, transventricular, and circumferential axis [40], as proposed in previous works [41,42], to obtain a physiological repolarization sequence, and in turn, a realistic T wave, in accordance with the signal morphology extracted from a large clinical ECG database [43]. Simulation parameters for the optimized QRST baseline experiment are given in Table S1. Based on these findings, the ventricular in silico experiments were repeated with adjusted V_1/2_ and gKs parameter values as described above. For each resulting noise-free 12-lead ECG, QT durations were calculated in a postprocessing step, as the time interval between the absolute signal amplitude in lead Einthoven II exceeded a threshold value of 0.001 mV for the first and the last time [44].

### 4.6. Data Analysis

All electrophysiology data were analyzed with Clampfit 10 (Molecular Devices, Sunnyvale, CA, USA), Origin (OriginLab Corp, Northampton, MS, USA), and Excel (Microsoft Corp, Seattle, WA, USA). Activation curves using the peak tail current, normalized to KCNQ1 wild-type or KCNQ1 wild-type with KCNE1, were analyzed to predict a gain- or loss-of-function for variant KCNQ1 and *I*_Ks_ channels. Voltage dependence of the activation of the *I*_Ks_ channel was determined by Boltzmann-fits from the tail currents of each variant, after normalizing to their maximum current, to investigate the half maximal activation. All values were expressed as means ± S.E.M.. Error bars in all figures represent S.E.M. values. Significance was assessed using two tailed Student’s T-test. Asterisks indicate significance: *, *p* < 0.05; **, *p* < 0.01; ***, and *p* < 0.001.

### 4.7. Ethics Statement

The study involving human tissue was conducted according to the guidelines of the Declaration of Helsinki, and approved by the Institutional Review Board (or Ethics Committee) of the University of München. Isolation of oocytes from *Xenopus laevis* toads was approved by the local ethics commission of the Regierungspräsidium Giessen (V54–19c 20 15 h 02 MR 20/28 Nr. A 4/2013).

## Figures and Tables

**Table 1:** Pathogenicity prediction of the different *KCNQ1* variants. PROVEAN (Protein Variation Effect Analyzer), PolyPhen-2 (prediction of functional effects of human nsSNPs), SIFT (Sorting Intolerant From Tolerant), and SNAP2 (predicting functional effects of sequence variants) tools were used. Predictions by PROVEAN protein software: score ≤ −2.5, deleterious; > −2.5, neutral. The Poly-Phen−2 score ranges from 0.0 (tolerated) to 1.0 (deleterious). The amino acid substitution prediction by SIFT is damaging when the score is ≤ 0.05, and tolerated if the score is > 0.05. SNAP2 predicts a score that ranges from −100 (strong neutral prediction) to +100 (strong effect prediction).

Mutant	PROVEAN (Score)	PolyPhen-2 (Score)	SIFT (Score)	SNAP2 (Score)
G119R	deleterious (−6.01)	possible damaging (0.691)	damaging (0.00)	effect (84)
F166del	deleterious (−13.9)			
G186_L187del	deleterious (−15.78)			
V254L	deleterious (−2.92)	probably damaging (0.959)	damaging (0.04)	effect (76)
L273V	deleterious (−2.92)	probably damaging (0.992)	tolerated (0.22)	neutral (−8)
K421E	neutral (−1.8)	possibly damaging (0.827)	tolerated (0.05)	effect (59)
R539L	deleterious (−5.73)	probably damaging (0.995)	tolerated (0.05)	effect (70)
R591C	deleterious (−5.73)	probably damaging (1)	damaging (0.00)	effect (88)
G430fs*28	deleterious (−4.4)			

## Data Availability

The data used to support the findings of this study are included in the article. In silico experiments were deposited in a research data repository: “https://dx.doi.org/10.35097/580 (29. March 2022)”.

## Supplementary Materials

The following supporting information can be downloaded at: https://www.mdpi.com/article/10.3390/ijms24021350/s1.
