# Supplementary material for: Functional Characterization of a Spectrum of Novel Romano-Ward Syndrome KCNQ1 Variants
Source: Int J Mol Sci. 2023 Jan 10;24(2):1350. doi: 10.3390/ijms24021350 (PMC9865342; doi:10.3390/ijms24021350)
Supplement: Supplementary file 1 [file ijms-24-01350-s001.zip › ijms-2097812-supplementary.pdf]

Supplementary Figure S1

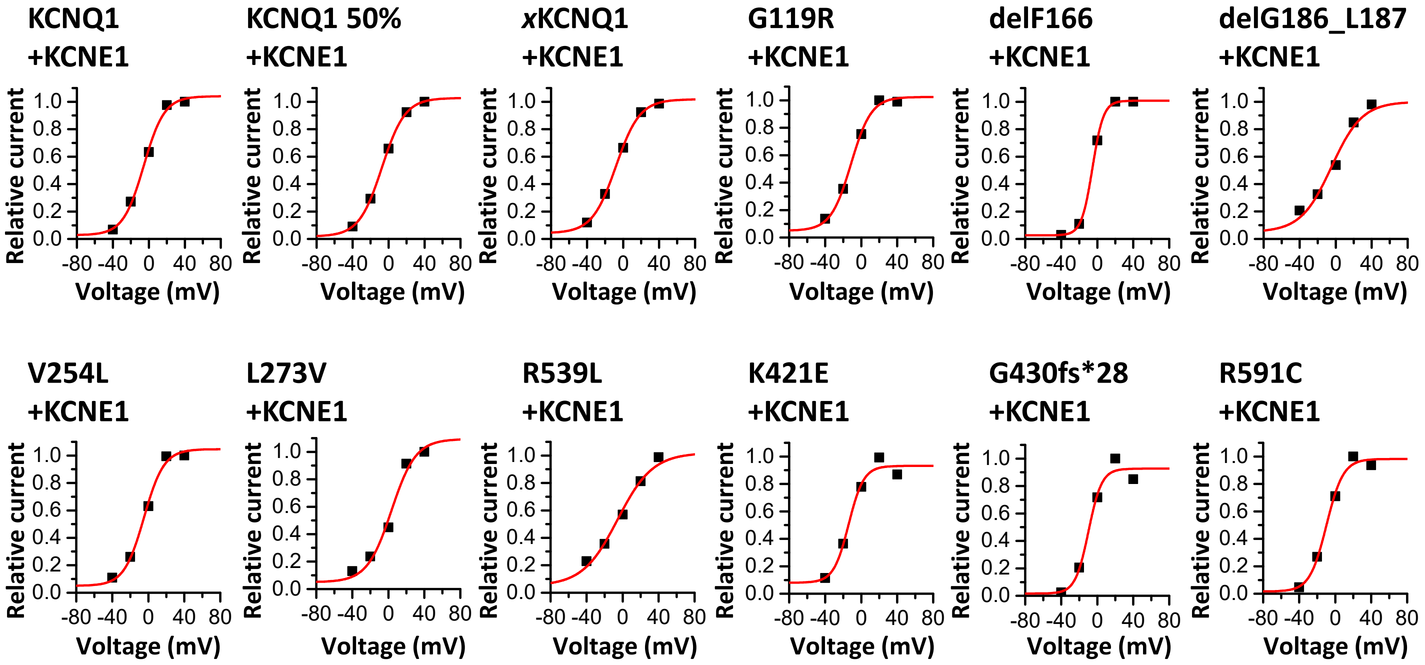

Supplementary Figure S1. Representative examples of the conductance-voltage (G/V) relationships for the different KCNQ1 variants. Recordings were performed with the protocol as described in Figure 6. The tail currents recorded after the 7 s pulse were normalized to the respective maximal tail current of each recording to obtain the conductance-voltage (G/V) curves. Normalized tail currents were fitted to a Boltzmann equation.

Supplementary Figure S2

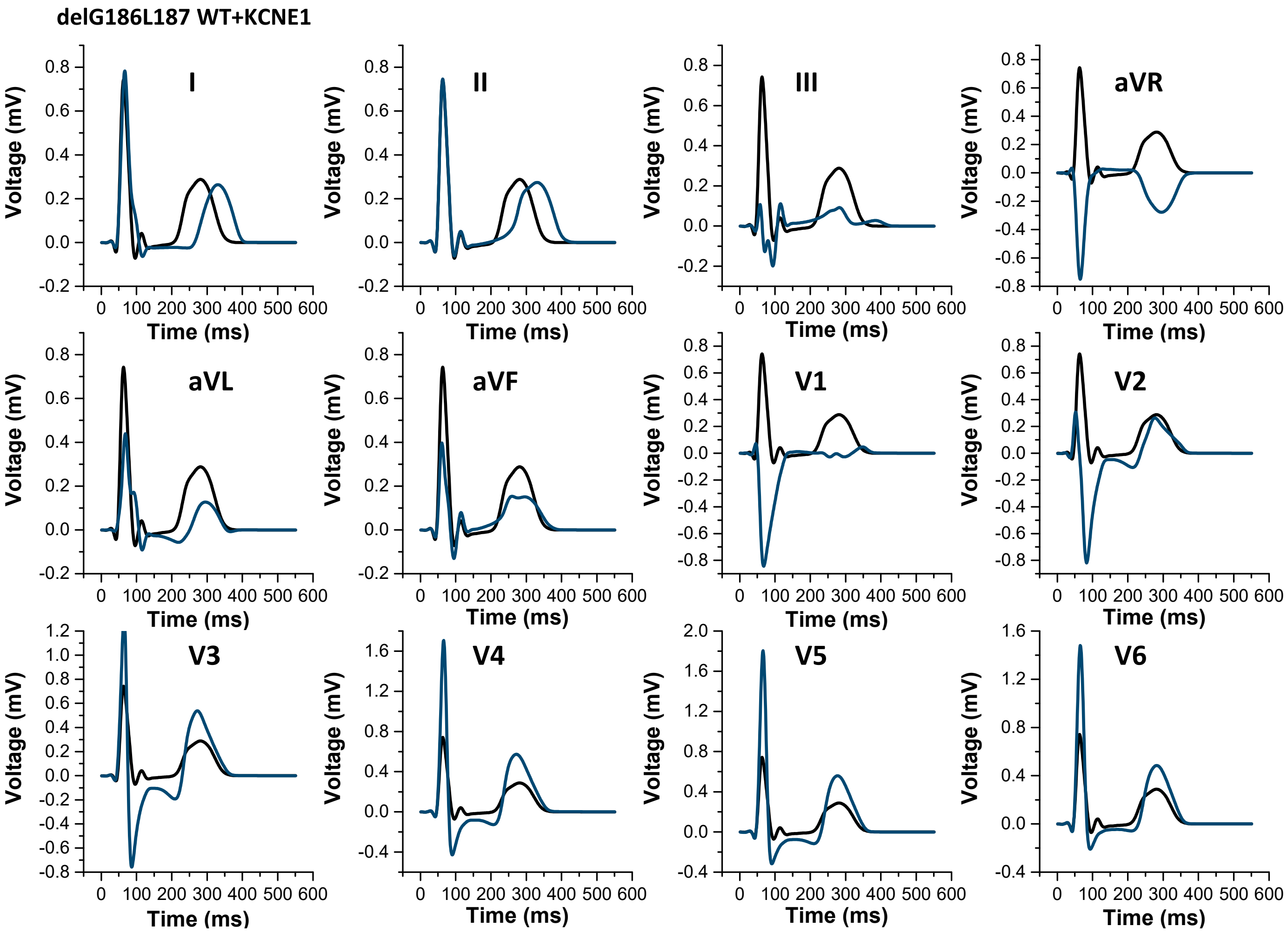

Supplementary Figure S2. 12-lead ECG modelling of wild-type KCNQ1+KCNE1 and the delG186_L187 variant. Lead I to lead V6 were calculated and plotted for wild-type KCNQ1 + KCNE1 (black) or the G186_187 variant (blue).

Supplementary Table S1: Simulation parameters for the *in silico* ECG computation. The scaling factors for the repolarization gradients were applied as described by Gillette *et al.* [1].

Feature	Parameter	Value
**Conduction velocities**	Transversal CV in myocardium	$CV_{m,t}$ = 0.3m/s
	Longitudinal CV in myocardium	$CV_{m,l}$ = 0.6m/s
	Transversal CV in subendocardial layer	$CV_{SE,t}$ = 1.1m/s
	Longitudinal CV in subendocardial layer	$CV_{SE,l}$ = 1.1m/s
**Initially activated regions**	Left ventricular anterior stimulus, Position in ventricular coordinates	$LV_{ant,ab}$ = 0.3042<br>$LV_{ant,rot}$= 0.4750
	Left ventricular anterior stimulus, delay	$LV_{ant,t}$ = 9.62ms
	Left ventricular anterior stimulus, radius	$LV_{ant,r}$ = 19.29mm
	Left ventricular posterior stimulus, Position in ventricular coordinates	$LV_{post,ab}$ = 0.6250<br>$LV_{post,rot}$= 0.1500
	Left ventricular posterior stimulus, delay	$LV_{post,t}$ = 4.25ms
	Left ventricular posterior stimulus, radius	$LV_{post,r}$ = 14.33mm
	Right ventricular stimulus, Position in ventricular coordinates	RV_{ab} = 0.4208<br>RV_{rot}= 0.7000
	Right ventricular stimulus, delay	RV_{t} = 0ms
	Right ventricular stimulus, radius	RV_{r} = 19.29mm
**Repolarization**	Minimum action potential duration at 90% repolarization	$APD_{90,min}$ = 215.29ms
	Maximum action potential duration at 90% repolarization	$APD_{90,max}$ = 381.36ms
	g_{Ks} scaling factor in apico-basal direction	$f_{gKs,ab}$ = 0.9957
	g_{Ks} scaling factor in transmural direction	$f_{gKs,tm}$ = 0.2276
	g_{Ks} scaling factor in transventricular direction	$f_{gKs,tv}$ = 0.5751
	g_{Ks} scaling factor in circumferential direction	$f_{gKs,rot}$ = 0.4956
**Forward calculation**	Torso conductivity	g_{Torso} = 0.2 S/m

1. Gillette, K.; Gsell, M. A. F.; Prassl, A. J.; Karabelas, E.; Reiter, U.; Reiter, G.; Grandits, T.; Payer, C.; Stern, D.; Urschler, M.; Bayer, J. D.; Augustin, C. M.; Neic, A.; Pock, T.; Vigmond, E. J.; Plank, G., A Framework for the generation of digital twins of cardiac electrophysiology from clinical 12-leads ECGs. *Medical image analysis* **2021,** 71, 102080.
